# Investigation of influential environmental and climatic determinants on COVID-19 spread in India to formulate a sustainable pandemic response

**DOI:** 10.1016/j.onehlt.2025.101042

**Published:** 2025-04-16

**Authors:** Jaraline Kirubavathy K., Thulasi Bai V.

**Affiliations:** a Research Scholar, Anna University, Faculty of Electronics and Communication Engineering, KCG College of Technology, Karapakkam, Chennai 600 097, India; b Professor, Faculty of Electronics and Communication Engineering, KCG College of Technology, Karapakkam, Chennai 600 097, India

**Keywords:** Pandemic preparedness, Pathogens, Sustainability, Environment, Climate change

## Abstract

The COVID-19 pandemic has highlighted the need for a Sustainable Pandemic Response Strategy (SPRS), driven by scientific research and engineering principles. This study focuses on Environmental and Climatic Determinants (ECDs) that may influence the occurrence pattern of infectious diseases. The objective of SPRS is to develop a climate-resilient framework for infectious diseases using Earth Observation (EO) data. ECDs were derived from EO data during the COVID-19 study period in India, spanning 1094 days (January 3, 2020, to December 31, 2022).

A Convergent Search – Add or Eliminate (CS-AE) algorithm was developed for the investigation of complex association between ECDs and disease occurrence patterns. This algorithm identifies the most influential ECDs in the spread of COVID-19 in India, categorizing them as Determinants of Concern (DOC) or Determinants of Interest (DOI). Shortwave Downward Radiation (SDR) was identified as a DOC, showing a strong correlation (*r* = 0.9525) with COVID-19 spread.

Granger causality analysis was conducted to support the classification of SDR as a Determinant of Concern (DOC). The results confirmed a temporal causal relationship between SDR and disease spread. During the first pandemic wave, significant causality was observed at lags of 2 to 7 days, with the strongest effect at lag 6 (*p* = 0.001), while in subsequent waves, significance was found across lags of 1 to 6 days. The seasonal effect of SDR and the three pandemic waves in India were observed through a radar chart, illustrating the temporal causal relationship between SDR and COVID-19 spread.

The algorithm shows the note of a significant role by SDR in surface and air temperature (*r* = 0.9525; *r* = 0.9942) and influences other ECDs which are categorized as DOI. Hence, the proposed CS-AE algorithm provides a robust tool for identifying the most influential ECDs in the spread of infectious diseases, provided the datasets are time-series based.

## 1 Introduction

The 21st century has seen the emergence and re-emergence of several infectious diseases, including SARS-CoV, MERS-CoV, and SARS-CoV-2—members of the coronavirus family that have posed recurring global health challenges. The COVID-19 pandemic, driven by SARS-CoV-2, demonstrated the widespread impact of emerging pathogens and underscored the need for proactive, data-driven public health strategies [1]. Understanding the spatial and temporal variation of infectious diseases is essential for improving early warning systems and outbreak preparedness.

### 1.1 From ecosystem crisis to one health

**Fig. 1:**
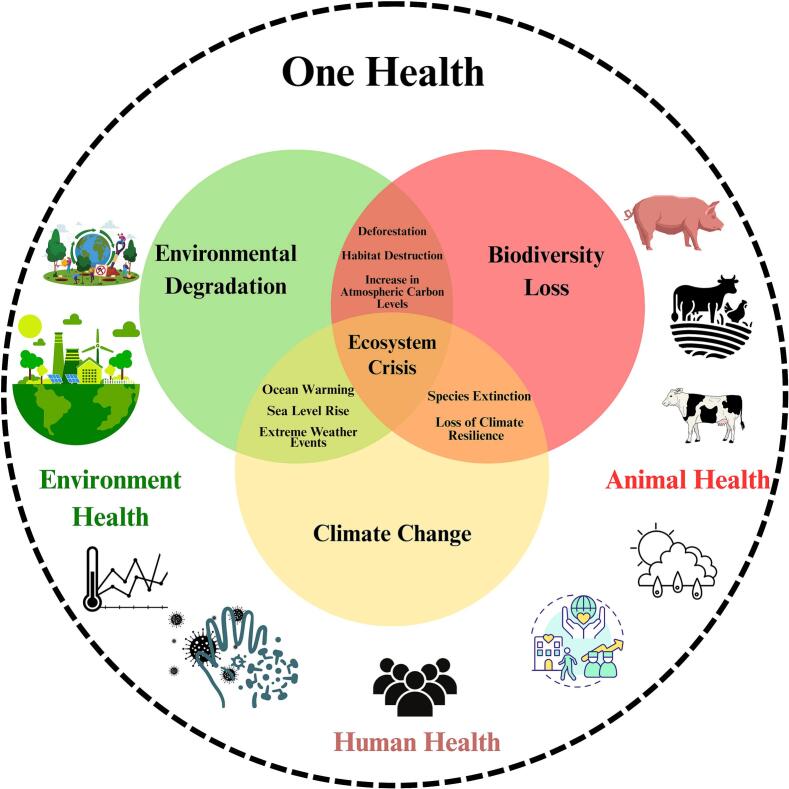
From ecosystem crisis to One Health.

Environmental degradation, biodiversity loss, and climate change interact to destabilize ecosystems, influencing the persistence and transmission of pathogens [2]. These stressors contribute to shifts in infectious disease patterns and, in densely populated areas, may enable the emergence of novel variants—as observed during the COVID-19 pandemic [[3], [4], [5], [6], [7], [8]]. As shown in Fig. 1, environmental degradation acts locally, biodiversity loss operates regionally to globally, and climate change exerts global influence. Their interconnected effects form a feedback loop that contributes to emerging diseases such as SARS, MERS, Ebola, Nipah and others [9,10].

Addressing these challenges requires a holistic One Health approach, grounded in understanding ecosystem crises through the analysis of Environmental and Climatic Determinants (ECDs) influencing disease dynamics [11,12].

### 1.2 Importance of creating sustainable pandemic response strategy (SPRS)and its purpose

World Health Organization released Pandemic Influenza Preparedness (PIP) Framework, for 2024–2030 which states that study of the impact of environment degradation and climate change becomes significant to contribute to human health and animal health. This shows the need for introspect of recent pandemic agent, SARS-CoV-2 with various ECDs [13]. To address this emerging concern, the development of Sustainable Pandemic Response Strategy (SPRS) is essential for addressing the future infectious disease challenges in the coming decades. The fundamental goal of SPRS is to develop climate-resilient infectious disease framework through a study of the association of ECDs with an infectious disease.

During the prevalence of COVID-19, WHO categorized the variant as Variant of Concern (VOC) and Variant of Interest (VOI) based on the severity of the occurrence pattern of COVID-19. For instance, studies have shown that VOCs such as Delta and Omicron exhibit surface stability that could influence transmission potential under various environmental conditions [14]. Moreover, climatic variables like temperature and humidity were found to significantly impact COVID-19 spread in different regions, emphasizing the role of environmental factors in modulating pandemic dynamics [[15], [16], [17], [18], [19], [20]]. Drawing from WHO's approach of classifying viral variants, this study proposes categorizing ECDs into:

• Determinants of Concern (DOCs): ECDs that exhibit a strong correlation with disease transmission and may also influence other ECDs.
• Determinants of Interest (DOIs): ECDs that are correlated with disease occurrence patterns and are likely influenced by DOCs.

This categorization helps provision of a framework of influential ECDs for an infectious disease irrespective of other confounding factors. This can help release of suitable polices and actions like lock down and travel restriction during outbreak or pandemic, and the effect of monitoring the concern ECDs and taking necessary precautionary steps to avoid the outbreak. Hence the proposed investigation is focused only on the investigation of the association of ECDs and the infectious disease, which will contribute to building the foundational knowledge needed for SPRS.

### 1.3 Limitations and gaps in existing research on COVID-19 and ECDs

Several studies have examined the correlation between COVID-19 occurrence and ECDs, primarily during the first or second pandemic waves. Meta-analyses report significant associations with temperature, humidity, wind speed, and population density. Early investigations in China, India, and the USA considered limited parameters, with around 517 articles focusing solely on temperature and humidity. Commonly identified ECDs include temperature, humidity, rainfall, dew point, sunshine duration, pressure, wind speed, and population density. Some studies found a negative correlation with temperature, while others reported a positive association with Diurnal Temperature Range (DTR). A few also explored UV radiation, solar radiation, and wind direction [[21], [22], [23], [24], [25]].

These ECDs have been examined using diverse analytical approaches, including correlation methods (Spearman, Kendall, and linear correlations), statistical and regression models (generalized additive models, negative binomial regression), causal and time-series models (Granger causality, vector autoregressive models, autoregressive distributed lag models), spatial analysis (bivariate spatial association), and machine learning techniques (e.g., artificial neural networks). Although COVID-19 transmission is influenced by various non-climatic factors such as socioeconomic and demographic variables, ECDs remain essential for understanding the environmental contribution to disease dynamics. Therefore, exploratory data analysis focusing on ECDs is vital for identifying their role in the spread of COVID-19 in India [[26], [27], [28]].

Despite the role of ECDs in shaping the spread of pathogens, the coronavirus remains a major threat due to its ability to drive recurring outbreaks and pandemics. This underscores the urgent need for dynamic epidemiological models that can identify outbreak patterns linked to seasonal or climatic variations. Developing such models will support evidence-based disease prevention strategies across different climates, especially in the absence of vaccines [29,30].

The survey highlights a research gap—most studies have not examined the association between COVID-19 and all available ECDs over the full pandemic period. Identifying influential ECDs across complete pandemic waves is essential for improving outbreak preparedness. There is also a need to standardize algorithms for detecting key ECDs in complex disease patterns to enhance predictive modeling and mitigation.

This study aims to develop a climate-resilient infectious disease framework by analysing the association between ECDs and COVID-19 spread. Beyond identifying correlated ECDs, the study seeks to uncover interacting or converging ones—termed DOCs. Since traditional statistical methods may not fully capture these complexities, the approach integrates evolutionary algorithms with correlation analysis to identify DOCs and DOIs requiring further validation.

## 2 Materials and methods

**Fig. 2:**
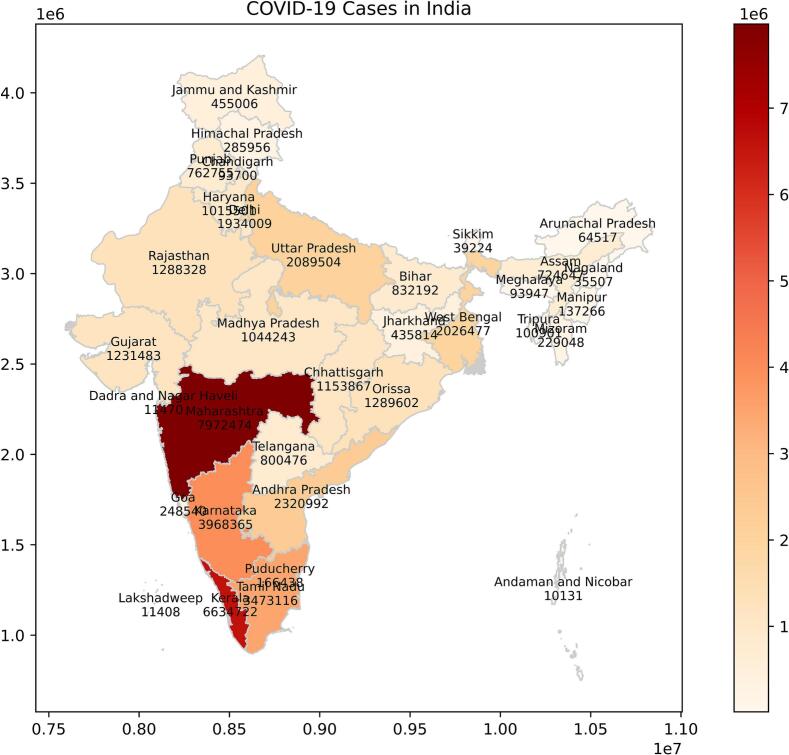
The geographical location of India.

India is the seventh largest geographic, and most populous country in the world. It is located at 20.5937°N and 78.9629°E. The geographical location of India, along with the occurrence rate of COVID-19 spread as of June 30, 2022, is shown in Fig. 2 (https://prsindia.org/covid-19/cases). As on May 2, 2023 India is the second highest confirmed COVID-19 cases in the world [26,33].

**Fig. 3:**
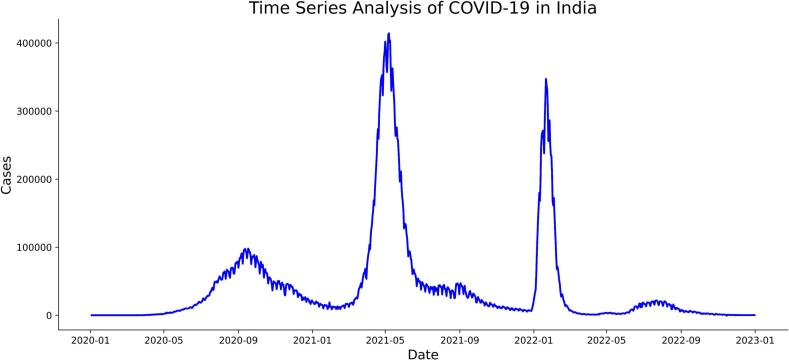
Time series analysis of COVID-19 occurrence in India.

Figure 3 shows the occurrence pattern of COVID-19 in India. India faced three pandemic waves during the year 2020–2022. The first wave that began in March 2020 subsided by November 2020. The second wave started in February 2021 and ended by June 2021. The third wave, driven by the Omicron variant, began in December 2021, surged in cases in early 2022, and started to decline by February 2022.

### 2.1 Spatial data preparation

Environmental time series data were obtained from Landsat 8 satellite imagery (LANDSAT/LC08/CO1/T1_TOA), which provides high-resolution observations with a spatial resolution of 15 to 30 m. Key environmental determinants such as NDVI, NDWI, EVI, and LST were derived using the Google Earth Engine (GEE) platform (https://code.earthengine.google.com/) from 2020 to 2022. GEE, a cloud-based geospatial tool, enables satellite image processing using JavaScript and Python through its API. Missing values in the environmental datasets were addressed using forward and backward fill imputation methods. Additional ECDs were retrieved from NASA's Prediction of Worldwide Energy Resources (POWER) portal (https://power.larc.nasa.gov/) for the COVID-19 period in India. As the NASA POWER data contained no missing values, it was directly used for analysing the impact of ECDs on COVID-19 spread. The temporal and spatial resolution of the Earth Observation data used is summarized in Table 1.

**Table 1:** Description of utilized environmental and climatic determinants (ECDs).

S.No.	Environmental and Climatic Determinants (ECDs)	Notation	Earth Observation Data
1	Land Surface Temperature	LST	Landsat 8 satellite images (LANDSAT/LC08/CO1/T1_TOA); Spatial and Temporal Resolution: 15-30 m and 16 days
2	Enhanced Vegetation Index	EVI	
3	Normalized Difference Wetness Index	NDWI	
4	Normalized Difference Vegetation Index	NDVI	
5	Precipitation	PP	
6	All Sky Surface Shortwave Downward Irradiance (MJ/m^2/day)	ASSDR	NASA POWER Spatial and Temporal Resolution: 0.5° (∼55 km) at the equator for surface data, 1° (∼111 km) for atmospheric data and, Daily, Monthly
7	Clear Sky Surface Shortwave Downward Irradiance (MJ/m^2/day)	CSSDR	
8	Top-Of-Atmosphere Shortwave Downward Irradiance (MJ/m^2/day)	TOASDR	
9	All Sky Surface PAR Total (W/m^2)	ASSPAR	
10	Clear Sky Surface PAR Total (W/m^2)	CSSPAR	
11	All Sky Surface UVA Irradiance (W/m^2)	ASSUVAR	
12	All Sky Surface UVB Irradiance (W/m^2)	ASSUVBR	
13	All Sky Surface UV Index (dimensionless)	ASSUVI	
14	Surface Soil Wetness	SSW	
15	Root Zone Soil Wetness	RZSW	
16	Profile Soil Moisture	PSM	
17	Temperature at 2 Meters (C)	T2M	
18	Dew/Frost Point at 2 Meters (C)	T2MDEW	
19	Wet Bulb Temperature at 2 Meters (C)	T2MWET	
20	Surface Temperature	TS	
21	Temperature at 2 Meters Range (C)	T2M_RANGE	
22	Temperature at 2 Meters Maximum (C)	T2M_MAX	
23	Temperature at 2 Meters Minimum (C)	T2M_MIN	
24	All Sky Surface Long-wave Downward Irradiance (W/m^2)	ASSLDR	
25	Specific Humidity at 2 Meters (g/kg)	QV2M	
26	Relative Humidity at 2 Meters (%)	RH2M	
27	Surface Pressure (kPa)	PS	
28	Wind Speed at 2 Meters (m/s)	WS2M	
29	Wind Direction at 2 Meters (Degrees)	WD2M	
30	Wind Speed at 10 Meters (m/s)	WS10M	
31	Wind Direction at 10 Meters (Degrees)	WD10M	

A total of 31 Environmental and Climatic Determinants (ECDs) were selected based on literature, focusing on factors frequently used in infectious disease monitoring. These determinants were chosen for their availability through freely accessible satellite and Earth observation datasets, enabling real-time monitoring and modeling of infectious disease dynamics through a diverse range of ECDs.

### 2.2 Proposed research framework for identifying influential ECDs

**Fig. 4:**
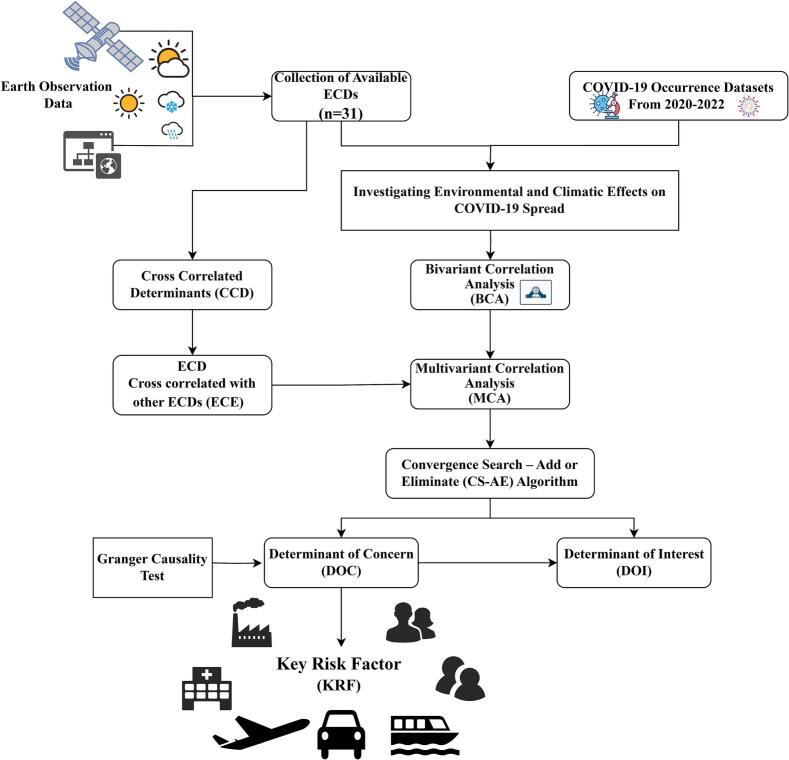
Proposed research framework.

Comprehensive exploration of direct, indirect, and complex associations is crucial for identifying influential ECDs that shape infectious disease dynamics. The proposed research framework shown as Fig. 4, provides the outline of the proposed investigation to identify influential ECDs and categorize ECDs as DOC and DOI. Direct associations between ECDs and COVID-19 spread were analyzed using Bivariate Correlation Analysis (BCA), indirect associations through Multivariate Correlation Analysis (MCA), and complex patterns via a correlation-based evolutionary algorithm. Influential ECDs were then identified and categorized as Determinants of Concern (DOCs) and Determinants of Interest (DOIs).

The proposed computational analysis has used Convergence Search – Add or Eliminate (CS-AE) algorithm. It comprises spatial data preparation, which includes the creation of an ECD table and a COVID-19 occurrence table, Bivariate Correlation Analysis (BCA), Creation of CCD (Cross Correlated Determinants) table, Creation of ECE (ECD Cross-correlated with other ECDs) table, Multivariate Cross-correlation Analysis (MCA), and finally, categorization of ECDs as DOC and DOI Convergence Search and Add or Eliminate (CS-AE) method.

The strength of association was evaluated using correlation coefficients (r). A value of ±1.0 indicates a perfect relationship where both variables consistently change together, while values between ±0.9 and ± 0.8 reflect a very strong association. Coefficients ranging from ±0.7 to ±0.6 indicate a moderate relationship, and values from ±0.5 to ±0.3 suggest a fair connection. A weak or poor association is represented by values between ±0.2 and ± 0.1, whereas a coefficient of 0 signifies no relationship between the variables [34,35].

**Fig. 5:**
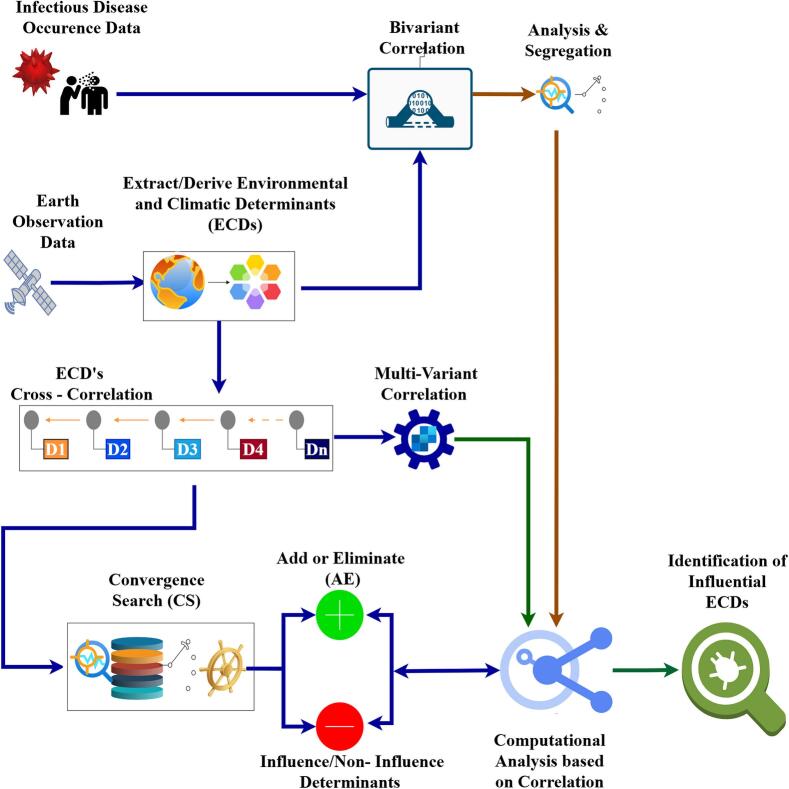
CS-AE Implementation Based on Evolutionary Steps.

The initial step in implementing the proposed research framework involves the collection of infectious disease occurrence data, along with the extraction and derivation of ECDs from Earth Observation (EO) data. Fig. 5 illustrates the steps for implementing the proposed CS-AE algorithm using correlation based evolutionary approach.

Based on the COVID-19 occurrence datasets and the extracted or derived ECDs from the EO data, create the following table to implement the proposed CS-AE algorithm.

#### 2.2.1 Create ECD table

Let $x_{\mathrm{ij}}$ and $y_{j}$ represents various ECD's and Covid-19 occurrence in India from 03 Jan 2020 to 31 Dec 2022.

$$x\in \left\{D_{i,1},D_{i,2},D_{i,3}\ldots ..,D_{i,n}\right\}$$

Where, $D_{1,}D_{2,}D_{3,}\ldots \mathrm{represents}\mathrm{various}\;\mathrm{EC}D'se\mathrm{racted}\mathrm{from}\mathrm{Earth}\mathrm{Observation}\mathrm{Data}\mathrm{and}n\mathrm{ranges}\mathrm{from}0\;\mathrm{to}\;31\;\mathrm{and}\;i\;\mathrm{ranges}\mathrm{from}\;1\;\mathrm{to}\;1094$.

#### 2.2.2 Create COVID-19 data table

Collect COVID-19 data from the portal and form a table as

$$C_{j}\in \left\{{\mathrm{CD}}_{1},{\mathrm{CD}}_{2},{\mathrm{CD}}_{3}\ldots ..,{\mathrm{CD}}_{j}\right\}$$

Where, $CD_{1,}CD_{2,}\ldots \mathrm{represents COVID}\;19\;\mathrm{occaranece rate},j\;\mathrm{rangesfrom}\;1\;\mathrm{to}\;1094$

#### 2.2.3 Create CCD (Cross Correlated Determinants) table

Perform cross correlation for the investigation of the complex linkage COVID-19 occurrence rate and ECDs, represent them as a Cross Correlated Determinants, CCD table.

#### 2.2.4 Create ECE (ECD Cross correlated with other ECDs) table - Selection

For each ECD, select the influential ECDs based on the correlation-based fitness function (*r* ≥ 0.6). Select only the moderate, strong and highly correlated ECDs with an ECD by using CCD table and create ECE table (ECD Cross correlated with other ECDs).

$${\mathrm{ECE}}_{n}\in \left\{{\mathrm{CCD}}_{1},{\mathrm{CC}D}_{2},{\mathrm{CCD}}_{3}\ldots .{\mathrm{CCD}}_{n}\right\}$$

Where, nranges from0≤n≤31

#### 2.2.5 Convergent Search (CS) process - crossover:

In this step, the algorithm searches for ECDs that exhibit significant interactions or convergence with one another. Accordingly, the relationships between each ECD and its cross-correlated counterparts are reassessed in every iteration to identify which determinants most strongly converge or interact with the spread of the disease. Each iteration is evaluated using a fitness function, with a correlation threshold of *r* ≥ 0.8. The outcome of the CS process is the identification of convergent or interactive determinants that satisfy this fitness criterion. The search process terminates when the selected ECDs meet or exceed the correlation threshold. If the threshold is not achieved, the process continues to optimize until the maximum possible correlation is obtained.

#### 2.2.6 Add or eliminate (AE) process – mutation

For each ECD, the algorithm evaluates its convergent ECDs, adding or eliminating non-influential and influential ones based on the fitness function (*r* ≥ 0.8). This process continues until a high correlation level is reached, ensuring that only the most influential ECDs are retained while overfitted ones are excluded from the final analysis.

Identification of Determinants of Concern (DOC) and Determinant of Interest (DOI) based on CS-AE method:

• *Determine DOC:* The DOC refer to ECD that play a significant role in the occurrence and spread of COVID-19. These determinants are often strongly correlated with disease transmission and may also influence other ECDs. DOCs are critical for explaining the spatial and temporal dynamics of COVID-19 and are essential to understanding and managing outbreaks. The identification of DOC is based on assessing the degree of involvement of each ECD, particularly through convergent/interactive effects and the add process incorporated in the CS-AE method.
• *Determine DOI:* After multiple iterations of selection, crossover, and mutation, the algorithm identifies a final set of ECDs that exhibit a very strong correlation with the occurrence pattern of COVID-19. ECDs with a correlation coefficient greater than 0.9 (*r* ≥ 0.9) are referred to as DOI, as they are considered to have the most significant impact on the transmission dynamics.

### 2.3 Granger causality test for validating DOC

The Granger causality test evaluates whether past values of one time series can predict future values of another, using lagged data. If the past values of variable X significantly improve the prediction of Y, then X is said to Granger-cause Y. The test provides both an F-test (for short-term, linear associations) and a Chi-square test (for long-term, potentially nonlinear associations), along with corresponding *p*-values. The ECDs showing statistical significance in these tests, as well as in correlation analysis, can be classified as DOC. Thus, the Granger causality test strengthens the identification of DOC by capturing temporal and causal relationships.

## 3 Implementation

The proposed CS-AE algorithm was implemented in RStudio version 3.6.0. Based on the extracted and derived ECDs using GEE API and COVID-19 occurrence rate, Bivariate Correlation Analysis (BCA) was performed using RStudio. This helped in understanding the level of direct association of COVID-19 spread and ECDs. Table 2 shows the result of the Bivariate Correlation Analysis (BCA) with COVID-19 occurrence rate and ECDs. Only the Enhanced Vegetation Index is moderately associated with the occurrence of COVID-19 in India.

**Table 2:** Bivariate correlation analysis (BCA).

S.No.	ECDs	Correlation	Severity Level
1	PP	0.3304	Fair Correlation
2	LST	0.5269	Fair Correlation
3	EVI	−0.6747	Moderate Correlation
4	NDWI	0.5382	Fair Correlation
5	NDVI	−0.4547	Fair Correlation
6	ASSDR	−0.1568	No Correlation
7	CSSDR	0.1961	No Correlation
8	TOASDR	0.2190	No Correlation
9	ASSPAR	−0.0809	No Correlation
10	CSSPAR	0.2007	No Correlation
11	ASSUVAR	−0.0355	No Correlation
12	ASSUVBR	0.0629	No Correlation
13	ASSUVI	0.1020	No Correlation
14	SSW	0.3102	Fair Correlation
15	RZSW	0.2986	No Correlation
16	PSM	0.2818	No Correlation
17	T2M	0.0765	No Correlation
18	T2MDEW	0.3085	Fair Correlation
19	T2MWET	0.2812	No Correlation
20	TS	0.046	No Correlation
21	T2M_RANGE	−0.3081	Fair Correlation
22	T2M_MAX	−0.0203	No Correlation
23	T2M_MIN	0.1763	No Correlation
24	ASSLDR	0.1942	No Correlation
25	QV2M	0.3088	Fair Correlation
26	RH2M	0.2319	No Correlation
27	PS	−0.2722	No Correlation
28	WS2M	0.0478	No Correlation
29	WD2M	0.1937	No Correlation
30	WS10M	0.0453	No Correlation
31	WD10M	0.1941	No Correlation

Out of 31 ECDs, EVI alone shows a moderate correlation with COVID-19 occurrence. A few determinants that include PP, LST, NDWI, NDVI, SSW, T2MDEW, T2M_RANGE, and QV2M exhibit a fair correlation. Notably, none of the parameters demonstrates a very strong correlation. Multivariate Correlation Analysis (MCA) is performed identify the indirect relationships between COVID-19 occurrence data and various ECDs.

### 3.1 Multivariate correlation analysis (MCA) – Indirect association analysis

Analysis of indirect influential ECDs on COVID-19 spread was performed using Multivariate Correlation Analysis (MCA). This is based on the Cross Correlated Determinants (CCD) for an each ECD with a correlation-based fitness function (*r* ≥ 0.6).

#### 3.1.1 Creation of cross correlated determinants (CCD)

**Fig. 6:**
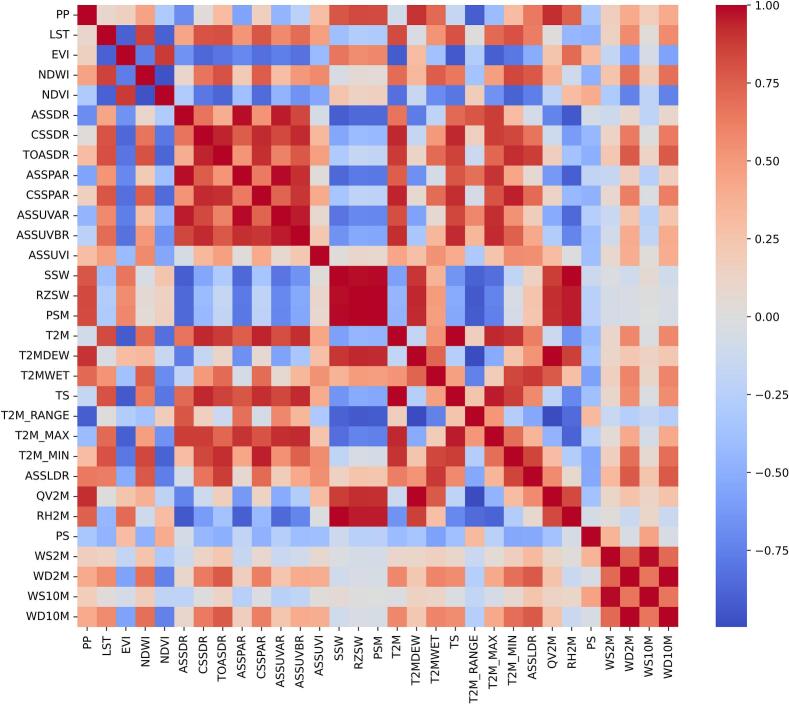
Cross correlated determinants (CCD) matrix.

To identify the association of one ECD with other ECDs, cross-correlation was performed using RStudio. This provides the Cross Correlated Determinants (CCD) table based on the Fig. 6 shows the CCD matrix depicting the relationship of one ECD with others.

#### 3.1.2 Creation of ECD cross correlated with other ECDs (ECE) table

Figure 5 illustrates how each ECD changes in relation to other ECDs through cross-correlation. To perform MCA, ECDs that exhibit perfect, very strong, moderate, and fair correlations (*r* ≥ 0.6) with other ECDs were extracted based on the fitness function. Table 3 shows the cross-correlated ECDs with each ECD. LST exhibits a moderate correlation only with EVI, whereas ASSUVI does not exhibit any significant correlation with other ECDs.

**Table 3:** ECD Cross correlated with other ECDs (ECE) table.

S.No.	ECDs	Cross Correlated ECDs
1	PP	SSW,RZSW,PSM,T2MDEW,T2M_RANGE, QV2M
2	LST	EVI
3	EVI	LST,NDWI, NDVI, TOASDR, T2M, TS,T2M_MAX, T2M_MIN
4	NDWI	EVI, NDVI
5	NDVI	EVI, NDWI, TOASDR
6	ASSDR	ASSPAR, ASSUVAR, ASSUVBR, T2M_MAX, RH2M
7	CSSDR	ASSPAR, ASSUVAR, ASSUVBR, T2M_MAX
8	TOASDR	EVI, NDVI, CSSDR, CSSPAR, ASSUVBR, T2M, T2MWET,TS, T2M_MAX, T2M_MIN, ASSLDR, WD2M, WD10M
9	ASSPAR	ASSDR, CSSDR, ASSUVAR, ASSUVBR, T2M_MAX, RH2M
10	CSSPAR	CSSDR, TOASDR, ASSUVBR, ASSUVAR, T2M, T2MWET, TS, T2M_MAX, T2M_MIN, ASSLDR, PS
11	ASSUVAR	ASSDR, CSSDR, ASSPAR, ASSUVBR, CSSPAR, T2M, TS, T2M_MAX
12	ASSUVBR	ASSDR, CSSDR, ASSPAR, CSSPAR, ASSUVAR, TOASDR, T2M, TS, T2M_MIN, T2M_MAX
13	ASSUVI	None
14	SSW	PP, RZSW, PSM, T2M_DEW, T2M_RANGE, T2M_MAX, QV2M, RH2M
15	RZSW	PP, SSW, PSM, T2M_DEW, T2M_RANGE, QV2M, RH2M
16	PSM	PP, SSW, RZSW, T2M_DEW, T2M_RANGE, QV2M, RH2M
17	T2M	EVI, CSSDR, TOASDR, CSSPAR, ASSUVAR, ASSUVBR, T2M_WET, TS, T2M_MAX, T2M_MIN, ASSLDR, PS
18	T2MDEW	PP, SSW, RZSW, PSM, T2M_WET, T2M_RANGE, ASSLDR, QV2M, RH2M
19	T2MWET	TOASDR, CSSPAR, T2M, T2M_DEW, T2M_RANGE, T2M_MIN, ASSLDR, QV2M, PS
20	TS	EVI, CSSDR, TOASDR, CSSPAR, ASSUVAR, ASSUVBR, T2M, T2M_MAX, T2M_MIN, ASSLDR
21	T2M_RANGE	PP, SSW, RZSW, PSM, T2M_DEW, T2M_WET, ASSLDR, QV2M, RH2M
22	T2M_MAX	EVI, ASSDR, CSSDR, TOASDR, ASSPAR, CSSPAR, ASSUVAR, ASSUVBR, SSW, T2M, TS, T2M_MIN, RH2M
23	T2M_MIN	EVI, CSSDR, TOASDR, CSSPAR, T2M, T2M_WET, TS, T2M_MAX, ASSLDR, PS, WD2M, WD10M
24	ASSLDR	TOASDR, CSSPAR, T2M, T2M_DEW, T2M_WET, TS, T2M_RANGE, T2M_MIN, QV2M
25	QV2M	PP, SSW, RZSW, PSM, T2M_DEW, T2M_WET, T2M_RANGE, ASSLDR, RH2M
26	RH2M	ASSDR, ASSPAR, SSW, RZSW, PSM, T2M_DEW, T2M_RANGE, T2M_MAX
27	PS	CSSPAR, T2M, T2M_WET, T2M_MIN, PS, WS2M, WS2M_MAX, WS2M_MIN, WS2M_RANGE, WS10M, WS10M_MIN, WS10M_MAX, WS10M_RANGE
28	WS2M	WS2M_MAX, WS2M_MIN, WS2M_RANGE, WS10M, WS10M_MIN, WS10M_MAX, WS10M_RANGE
29	WD2M	TOASDR, T2M_MIN, WD10M
30	WS10M	WS2M, WS2M_MIN, WS2M_MAX, WS2M_RANGE
31	WD10M	TOASDR, T2M_MIN, WD10M

Based on the ECE table performed MCA to explore the indirect association between the occurrence of COVID-19 and the ECDs and it shown in Table 4.

**Table 4:** Multivariate correlation analysis (MCA).

S.No.	ECDs	Cross Correlated ECDs (CCDs)	Correlation	Correlation Level
1	PP	SSW,RZSW,PSM,T2MDEW,T2M_RANGE, QV2M	0.2908	No Correlation
2	LST	EVI	0.5345	Fair Correlation
3	EVI	LST,NDWI, NDVI, TOASDR, T2M, TS,T2M_MAX, T2M_MIN	0.6047	Moderate Correlation
4	NDWI	EVI, NDVI	0.4856	Fair Correlation
5	NDVI	EVI, NDWI, TOASDR	0.5041	Fair Correlation
6	ASSDR	ASSPAR, ASSUVAR, ASSUVBR, T2M_MAX, RH2M	0.2385	No Correlation
7	CSSDR	ASSPAR, ASSUVAR, ASSUVBR, T2M_MAX	0.2064	No Correlation
8	TOASDR	EVI, NDVI, CSSDR, CSSPAR, ASSUVBR, T2M, T2MWET, TS, T2M_MAX, T2M_MIN, ASSLDR, WD2M, WD10M	0.5806	Fair Correlation
9	ASSPAR	ASSDR, CSSDR, ASSUVAR, ASSUVBR, T2M_MAX, RH2M	0.2459	No Correlation
10	CSSPAR	CSSDR, TOASDR, ASSUVBR, ASSUVAR, T2M, T2MWET, TS, T2M_MAX, T2M_MIN, ASSLDR, PS	0.3479	Fair Correlation
11	ASSUVAR	ASSDR, CSSDR, ASSPAR, ASSUVBR, CSSPAR, T2M, TS, T2M_MAX	0.3047	Fair Correlation
12	ASSUVBR	ASSDR, CSSDR, ASSPAR, CSSPAR, ASSUVAR, TOASDR, T2M, TS, T2M_MIN, T2M_MAX	0.3746	Fair Correlation
13	ASSUVI	No Correlation	–	None
14	SSW	PP, RZSW, PSM, T2M_DEW, T2M_RANGE, T2M_MAX, QV2M, RH2M	0.3326	Fair Correlation
15	RZSW	PP, SSW, PSM, T2M_DEW, T2M_RANGE, QV2M, RH2M	0.3286	Fair Correlation
16	PSM	PP, SSW, RZSW, T2M_DEW, T2M_RANGE, QV2M, RH2M	0.3286	Fair Correlation
17	T2M	EVI, CSSDR, TOASDR, CSSPAR, ASSUVAR, ASSUVBR, T2M_WET, TS, T2M_MAX, T2M_MIN, ASSLDR, PS	0.8669	Very Strong Correlation
18	T2MDEW	PP, SSW, RZSW, PSM, T2M_WET, T2M_RANGE, ASSLDR, QV2M, RH2M	0.3557	Fair Correlation
19	T2MWET	TOASDR, CSSPAR, T2M, T2M_DEW, T2M_RANGE, T2M_MIN, ASSLDR, QV2M, PS	0.3854	Fair Correlation
20	TS	EVI, CSSDR, TOASDR, CSSPAR, ASSUVAR, ASSUVBR, T2M, T2M_MAX, T2M_MIN, ASSLDR	0.5825	Fair Correlation
21	T2M_RANGE	PP, SSW, RZSW, PSM, T2M_DEW, T2M_WET, ASSLDR, QV2M, RH2M	0.3557	Fair Correlation
22	T2M_MAX	EVI, ASSDR, CSSDR, TOASDR, ASSPAR, CSSPAR, ASSUVAR, ASSUVBR, SSW, T2M, TS, T2M_MIN, RH2M	0.6098	Moderate Correlation
23	T2M_MIN	EVI, CSSDR, TOASDR, CSSPAR, T2M, T2M_WET, TS, T2M_MAX, ASSLDR, PS, WD2M, WD10M	0.57907	Fair Correlation
24	ASSLDR	TOASDR, CSSPAR, T2M, T2M_DEW, T2M_WET, TS, T2M_RANGE, T2M_MIN, QV2M	0.3694	Fair Correlation
25	QV2M	PP, SSW, RZSW, PSM, T2M_DEW, T2M_WET, T2M_RANGE, ASSLDR, RH2M	0.3557	Fair Correlation
26	RH2M	ASSDR, ASSPAR, SSW, RZSW, PSM, T2M_DEW, T2M_RANGE, T2M_MAX	0.6445	Moderate Correlation
27	PS	CSSPAR, T2M, T2M_WET, T2M_MIN, PS, WS2M, WS2M_MAX, WS2M_MIN, WS2M_RANGE, WS10M, WS10M_MIN, WS10M_MAX, WS10M_RANGE	0.1907	No Correlation
28	WS2M	WS2M_MAX, WS2M_MIN, WS2M_RANGE, WS10M, WS10M_MIN, WS10M_MAX, WS10M_RANGE	0.1823	No Correlation
29	WD2M	TOASDR, T2M_MIN, WD10M	0.1329	No Correlation
30	WS10M	WS2M, WS2M_MIN, WS2M_MAX, WS2M_RANGE	0.1823	No Correlation
31	WD10M	TOASDR, T2M_MIN, WD10M	0.1329	No Correlation

It shows, Air Temperature (*r* = 0.8669) exhibits a very strong association with COVID-19 occurrence in India. A few determinants such as Enhanced Vegetation Index (*r* = 0.6047), Maximum Temperature (*r* = 0.6098), and Relative Humidity (*r* = 0.6445) exhibit a moderate association. The BCA revealed no direct influential ECD for COVID-19 spread, whereas MCA identified Air Temperature as an indirect influential ECD. The complex interplay between ECDs and COVID-19 spread can be further investigated using the proposed CS-AE algorithm. This investigation helps categorization of the ECDs as DOC and DOI.

## 4 Results and discussion

The CS-AE method was implemented in R Studio to investigate complex associations. As shown in Table 5, twenty ECDs exhibited a very strong association with COVID-19 occurrence, while seven demonstrated moderate and three showed fair levels of association.

**Table 5:** Convergence search – add or eliminate (CS-AE) algorithm.

S.No.	ECDs	Convergent Determinants (CDs)	Added Determinant	Eliminated Determinant	Correlation	Correlation Level
1	PP	QV2M,TOASDR	TOASDR	–	0.7914	Moderate Correlation
2	LST	EVI,TOASDR	TOASDR		0.7979	Moderate Correlation
3	EVI	CSSDR,TOASDR	–	T2M_MIN	0.9658	Very strong Correlation
4	NDWI	EVI,TOASDR	–	NDVI	0.7820	Moderate Correlation
5	NDVI	EVI,TOASDR	–	NDVI	0.7820	Moderate Correlation
6	ASSDR	ASSDR,TOASDR	–	T2M_MAX	0.8553	Very Strong Correlation
7	CSSDR	T2M_MAX,TOASDR	–	–	0.7565	Moderate Correlation
8	TOASDR	TOASDR,CSSPAR	–	–	0.9525	Very Strong Correlation
9	ASSPAR	TS,T2M_MAX	–	–	0.9027	Very Strong Correlation
10	CSSPAR	ASSUVAR,ASSUVBR	TOASDR		0.9486	Very Strong Correlation
11	ASSUVAR	ASSLDR,PS	–	–	0.8612	Very Strong Correlation
12	ASSUVBR	T2M,T2M_MAX			0.8007	Very Strong Correlation
13	ASSUVI	–	–	–	–	
14	SSW	TS,T2M_MAX	TOASDR	–	0.9759	Very Strong Correlation
15	RZSW	T2MDEW,T2M_RANGE			0.8778	Very Strong Correlation
16	PSM	T2M_MAX,TOASDR			0.8778	Very Strong Correlation
17	T2M	T2MDEW,T2M_RANGE			0.9942	Very Strong Correlation
18	T2MDEW	T2MDEW,T2M_RANGE			0.9942	Very Strong Correlation
19	T2MWET	CSSPAR,TOASDR			0.8419	Very Strong Correlation
20	TS	T2M_MAX,ASSLDR			0.9526	Very Strong Correlation
21	T2M_RANGE	T2MWET,T2M_RANGE	TOASDR	ASSLDR	0.9237	Very Strong Correlation
22	T2M_MAX	T2M_MAX,T2MDEW			0.9445	Very Strong Correlation
23	T2M_MIN	EVI,TOASDR			0.9940	Very Strong Correlation
24	ASSLDR	RH2M,TOASDR			0.8890	Very Strong Correlation
25	QV2M	CSSDR,TOASDR		ASSLDR	0.9224	Very Strong Correlation
26	RH2M	ASSUVAR,ASSUVBR			0.7704	Moderate Correlation
27	PS	TOASDR,CSSPAR		T2MWET,T2MMIN	0.8492	Very Strong Correlation
28	WS2M	T2M_RANGE,T2M_MIN	TOASDR	WS2M,WS2M_MAX,	0.6408	Moderate Correlation
29	WD2M	T2MWET,T2M_RANGE	EVI	WS2M_MIN, WS2_RANGE,WS10M	0.5967	Fair Correlation
30	WS10M	RH2M,ASSDR	EVI,TOASDR	WS10M_MIN, WS10M_MAX,	0.5392	Fair Correlation
31	WD10M	T2M_RANGE,T2M_MAX	EVI	WS10M_RANGE	0.5967	Fair Correlation

### 4.1 Significant findings from CS-AE algorithm

The CS-AE algorithm has demonstrated a robust capacity for the identificaiton and categorization of ECDs, their interactions with Convergent Determinants (CDs) and the occurrence pattern of an infectious disease. This helps unravelling the intricate dynamics between different climatic variables, offering valuable insights for understanding environmental factors influencing occurrences of infectious diseases like COVID-19. The algorithm highlights the convergence of multiple ECDs, that point to a collective influence on disease occurrence. Some of key findings of the proposed CS-AE algorithm are:

1. Land Surface Temperature (*r* = 0.7979) is a key ECD linked with the Enhanced Vegetation Index and Top-of-Atmosphere Shortwave Downward Irradiance. These interactions reveals the interplay between temperature, vegetation, and radiative energy, which collectively influence the overall climate.
2. Precipitation (*r* = 0.7914) is associated with specific humidity and top-of-atmosphere shortwave downward irradiance, highlighting the combined effect of moisture and radiative heat on climate and potential health outcomes.
3. Normalized Difference Wetness Index (*r* = 0.7820) and Normalized Difference Vegetation Index (r = 0.7820) are tied to EVI and TOASDR with the underlining of the relationship between vegetation, moisture levels, and shortwave radiation.
4. Shortwave Downward Radiation (r = 0.9525) and Long-wave Downward Irradiance (*r* = 0.8890), interact in complex ways for the influence on surface temperatures, humidity, and atmospheric pressure. These components affect both direct climatic conditions and indirect feedback mechanisms (e.g., disease transmission).
5. Temperature (*r* > 0.8) variables (T2M_MAX, T2M_MIN, T2M, T2MDEW, T2M_RANGE) exhibit intricate links with various environmental factors, such as Wet Bulb Temperature (T2MWET), Dew Point (T2MDEW), and Specific Humidity (QV2M). These interactions are significant in understanding the impact of temperature fluctuations on public health, including the incidence of COVID-19.
6. Top-Of-Atmosphere Shortwave Downward Irradiance (*r* = 0.9525), as a central determinant, is frequently identified as interacting with other key variables such as CSSPAR, ASSDR, and T2M_MAX. The synergy between these factors indicates that changes in one element, such as radiation levels, can impact multiple atmospheric variables with further affecting the overall climate system. TOASDR interacts with variables such as EVI, QV2M, and T2M, revealing the influence of radiation directly on surface temperatures, moisture content, and humidity. These factors have a significant bearing on disease transmission patterns, as variations in these climatic conditions can create conducive environments for viral spread. To conclude, Shortwave Downward Radiation (SDR) is shown as a Core Determinant among other determinants.
7. All Sky Surface PAR (*r* = 0.9027) is linked with temperature variables like T2M_MAX, T2M_MIN, and TS (surface temperature), demonstrating the contribution between light intensity and temperature for the influence of combine climate patterns and potentially impact disease occurrence.
8. Air temperature (r = 0.9942) and dew point temperature (r = 0.9942) have a significant relationship with humidity levels (QV2M and RH2M), influencing the air's capacity to hold moisture and potentially affecting viral stability and transmission.
9. Surface Pressure (*r* = 0.8492) interacts with TOASDR and CSSPAR, with influence on atmospheric dynamics and contributing to feedback loops that affect climate and health outcomes.
10. Soil moisture indices such as Surface Soil Wetness (*r* = 0.9759) and Root Zone Soil Wetness (*r* = 0.8778), and vegetation-related indices like NDVI and EVI, are critical in understanding the ecological environment's role in disease spread. Vegetation, soil moisture, and radiative energy together impact surface temperatures and humidity, thus playing an indirect role in shaping the environment's susceptibility to outbreaks.

Table 6 shows ECDs with very strong correlation (r ≥ 0.9). Out of 31 ECDs, twenty exhibited a very strong and complex association. Hence these ECDs are categorized as DOI based on CS-AE algorithm.

**Table 6:** Determinants of interest (DOI) based on CS-AE algorithm.

S.No.	ECDs	Correlation Level	Convergent Determinant
1	EVI	Very Strong	Shortwave Downward Radiation
2	ASSDR	Very Strong	Shortwave Downward Radiation
3	TOASDR	Very Strong	Photosynthetically Active Radiation
4	ASSPAR	Very Strong	Surface Temperature
5	CSSPAR	Very Strong	Shortwave Downward Radiation
6	ASSUVAR	Very Strong	Longwave Downward Radiation, Surface Pressure
7	ASSUVBR	Very Strong	Temperature
8	SSW	Very Strong	Shortwave Downward Radiation, Surface Temperature
9	RZSW	Very Strong	Air Temperature
10	PSM	Very Strong	Shortwave Downward Radiation, Surface Temperature
11	T2M	Very Strong	Dew Point and Air Temperature
12	T2MDEW	Very Strong	Air Temperature
13	T2MWET	Very Strong	Shortwave Downward Radiation, Photosynthetically Active Radiation
14	TS	Very Strong	Longwave Downward Radiation and Air Temperature
15	T2M_RANGE	Very Strong	Shortwave Downward Radiation, Wet-Bulb Temperature
16	T2M_MAX	Very Strong	Dew Point Temperature
17	T2M_MIN	Very Strong	Shortwave Downward Radiation
18	ASSLDR	Very Strong	Shortwave Downward Radiation, Relative Humidity
19	QV2M	Very Strong	Shortwave Downward Radiation
20	PS	Very Strong	Shortwave Downward Radiation, Photosynthetically Active Radiation

It also reveals Shortwave Downward Radiation (SDR) as a predominant influential ECD, because it significantly impacting several other ECDs.

### 4.2 Determinant of concern (DOC) for the occurrence pattern of COVID-19

In the investigation of influential ECDs on COVID-19 spread using the CS-AE algorithm, SDR was identified as a predominant influential ECD. The radar chart facilitates visual comparison between multiple quantitative variables across different categories. It also highlights patterns, trends, and relationships in multivariate data and helps getting the complex information.

**Fig. 7:**
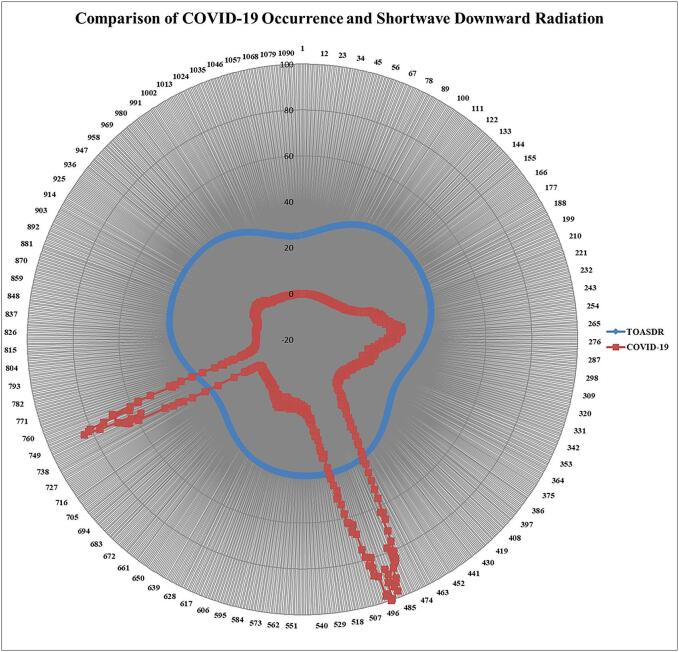
Comparison of top of atmosphere shortwave downward radiation (TOASDR) and COVID-19 occurrences in India.

Figure 7 showcases a radar chart, illustrating the relationship between COVID-19 occurrences and shortwave downward radiation. India experienced three significant spikes in COVID-19 cases during the pandemic, aligning closely with variations in shortwave downward radiation, identified as a concern determinant influencing the spreading dynamics of SARS-CoV-2 pathogen. The discrepancy in the spike timing between TOASDR and COVID-19 occurrences could be by two possibilities: (1) Delays in recording the dates when COVID-19 cases were reported: This means that there may be delays between when individuals actually contracted the virus and when their cases were officially recorded in the data. (2) Delays in the appearance of symptoms associated with the SARS-CoV-2 virus: This refers to the time taken for individuals who have contracted the virus to begin experiencing symptoms. These delays in symptom onset can vary from person to person and may affect the timing of when cases are officially reported.

Variations in radiation levels have a direct or indirect effect on various ECDs with influence on the infectiousness of the SARS-CoV-2 pathogen. The overall trend shows three distinct phases of both rising and falling shortwave downward radiation alongside COVID-19 occurrences in India. This highlights the dynamic interplay between ECDs and the transmission of COVID-19.

**Fig. 8:**
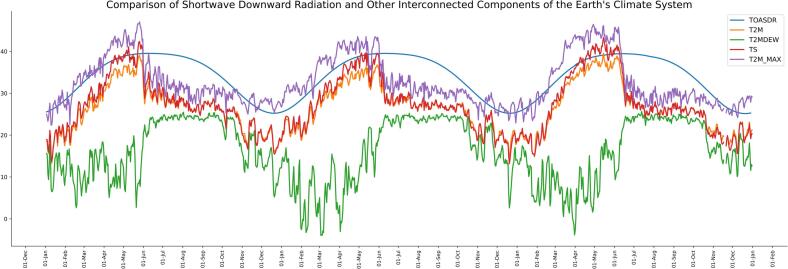
Comparison of shortwave downward radiation and other interconnected components of the earth's climate system.

Shortwave downward radiation serves as a pivotal factor in shaping atmospheric dynamics and impacting key elements such as air temperature, humidity, surface pressure, and surface temperature. These components form interconnected parts of the Earth's climate system, where alterations in one element can trigger ripple effects across others, creating intricate feedback loops and climate patterns. Therefore, in addition to shortwave downward radiation, as per the result of MCA, temperature plays a significant role in the occurrence pattern of COVID-19. Fig. 8, shows the comparison between trends and patterns of shortwave downward radiation and all forms of highly correlated temperature as per CS-AE algorithm such as dew temperature (T2MDEW), air temperature (T2M), and wet bulb temperature (T2MWET). The increasing and decreasing trend of air wet bulb and dew temperature is in correlated with SDR.

Using CS-AE algorithm, SDR was identified as a DOC. The highly correlated determinants (20 ECDs), namely, temperature, humidity, vegetation index, etc., are identified as DOI.

### 4.3 Granger causality test to claim DOC

The SDR was found to be a DOC, which may be a KRF when it studied with other confounding factors. The identified DOC can be investigated further through Granger causality test to strengthen the claim.

#### 4.3.1 Granger causality test for DOC

The Granger causality test between SDR and the COVID-19 occurrence rate was performed to strengthen the claim of DOC. This test was employed to identify potential temporal causality between SDR and the spread of COVID-19. It was conducted for the occurrence of three different pandemic waves in India.

Table 8 presents two tests, namely the F-test and the chi-square test, along with their significance values (p-values).

**Table 8:** Granger causality test for DOC – shortwave downward radiation.

ECDs	Lag	First Pandemic Wave				Second Pandemic Wave				Third Pandemic Wave			
		F-Test		Chi2 test		F-Test		Chi2 test		F-Test		Chi2 test	
		F	p	chi2	p	F	p	chi2	p	F	p	chi2	p
TOASDR	1	2.5743	0.1112	2.6387	0.1043	4.8438	0.0297	4.9659	0.0259	8.6489	0.0042	8.9472	0.0028
	2	3.7298	0.0269	7.7785	0.0205	5.1632	0.0071	10.7715	0.0046	9.4622	0.0002	29.3334	0
	3	3.0402	0.0319	9.6807	0.0215	4.9738	**0.0028**	15.8456	**0.0012**	9	**0**	29.3334	**0**
	4	2.4079	0.0536	10.4127	0.034	2.8926	0.0255	12.5171	0.0139	4.0024	0.0053	17.8568	0.0013
	5	2.4417	0.0388	13.4517	0.0195	2.1394	0.0662	11.7966	0.0377	3.327	0.0091	19.0748	0.0019
	6	5.0735	0.0001	34.2101	0	2.1051	0.0588	14.2097	0.0274	3.1024	0.0093	21.9753	0.0012
	7	8.2727	**0**	66.4246	**0**	1.0509	0.4009	8.4486	0.2947	1.7885	0.1035	15.2414	0.033
	8	0.6914	0.6983	6.4808	0.5935	1.2198	0.2955	11.4516	0.1774	1.6268	0.1341	16.3671	0.0374
	9	0.4219	0.9205	4.5484	0.8718	1.4261	0.1879	15.4016	0.0805	2.6369	0.0117	30.8897	0.0003
	10	0.4387	0.9238	5.3773	0.8646	1.4204	0.1838	17.4457	0.0651	1.8077	0.0786	24.4035	0.0066

For the first pandemic wave, TOASDR exhibits a temporal and causal relationship with the occurrence of COVID-19 at lags of 2, 3, 6, and 7. These lags may be attributed to delays in reporting the incidence rate, data collection, or the virus's incubation period before case detection. Such time delays are common in epidemiological data. Lag 2 might reflect an early influence of TOASDR on the spread, while lags 6 and 7 could indicate more prolonged effects, potentially due to reporting delays or longer incubation periods.

For the second pandemic wave, TOASDR shows a causal relationship with the occurrence of COVID-19 at lags of 1, 2, 3, and 4, indicating an immediate effect of TOASDR on COVID-19 incidence with shorter lags. This could suggest a quicker response or a more direct influence during this wave, possibly due to improved reporting or a reduced delay in case detection compared to the first wave.

For the third wave, the effect of TOASDR extends to lags of 1, 2, 3, 4, 6, and 7, suggesting a persistent influence of TOASDR for up to six days. It is reasonable to assume that the pandemic had reached a more complex stage at this point, with various factors (e.g., vaccine rollout, policy changes, public behaviour) affecting the speed and nature of case reporting and the spread of the virus.

#### 4.3.2 Granger causality test for SDRs interconnected components of earth's climate system

The interconnected components of Earth's climate system, influenced by Surface Downward Radiation (SDR), include air temperature, wet bulb temperature, dew point temperature, and surface temperature. In multivariate correlation analysis, air temperature was found to be highly correlated with the spread of COVID-19.

In the CS-AE algorithm, SDR was identified as playing a predominant role in COVID-19 spread. Additionally, air temperature (T2M), surface temperature (TS), wet bulb temperature (T2MWET), and dew point temperature (T2MDEW) were found to be interconnected components of SDR, exhibiting a strong correlation with COVID-19 spread according to the proposed approach.

Table 9 presents the Granger Causality Test for SDR's interconnected components of Earth's climate system. Air temperature (T2M) exhibited the most significant p-value for both the F-test and the chi-squared test. T2M showed a temporal and causal relationship at lag 7 during the first wave and lag 3 during the second and third pandemic waves.

**Table 9:** Granger causality test for SDRs interconnected components of earth's climate system.

ECDs	Lag	First Pandemic Wave				Second Pandemic Wave				Third Pandemic Wave			
		F-Test		Chi2 test		F-Test		Chi2 test		F-Test		Chi2 test	
		F	p	chi2	p	F	p	chi2	p	F	p	chi2	p
T2M	1	1.7723	0.1856	1.8167	0.1777	4.8438	0.0297	4.9659	0.0259	0.0235	0.8784	0.0243	0.876
	2	1.2584	0.2879	2.6243	0.2692	5.1632	0.0071	10.7715	0.0046	0.8563	0.4284	1.8146	0.4036
	3	1.8901	0.1352	6.0186	0.1107	4.9738	**0.0028**	15.8456	**0.0012**	3.0776	**0.0321**	10.0308	**0.0183**
	4	1.6787	0.1599	7.2594	0.1228	2.8926	0.0255	12.5171	0.0139	2.4668	0.0517	11.0058	0.0265
	5	1.8378	0.1115	10.1249	0.0718	2.1394	0.0662	11.7966	0.0377	2.0893	0.076	11.9786	0.0351
	6	2.4521	0.0293	16.5341	0.0112	2.1051	0.0588	14.2097	0.0274	2.0788	0.0662	14.7245	0.0225
	7	2.6313	**0.0153**	21.1278	**0.0036**	1.0509	0.4009	8.4486	0.2947	2.6374	0.0178	22.4754	0.0021
	8	1.2899	0.2575	12.0916	0.1472	1.2198	0.2955	11.4516	0.1774	1.5692	0.1512	15.7871	0.0455
	9	1.5893	0.1293	17.1346	0.0466	1.4261	0.1879	15.4016	0.0805	1.2903	0.26	15.1153	0.0878
	10	1.3543	0.214	16.6014	0.0837	1.4204	0.1838	17.4457	0.0651	1.0939	0.3815	14.7674	0.1408
T2MDEW	1	1.8426	0.1772	1.8887	0.1693	12.8436	**0.0005**	13.1674	**0.0003**	13.2625	**0.0005**	13.7198	**0.0002**
	2	0.9382	0.3943	1.9566	0.376	3.9695	0.0215	8.2813	0.0159	2.7012	0.073	5.7239	0.0572
	3	1.7358	0.1636	5.527	0.137	3.1035	0.0295	9.8872	0.0195	4.7842	0.004	15.593	0.0014
	4	1.6568	0.1651	7.1646	0.1274	1.925	0.1113	8.33	0.0802	2.364	0.0602	10.547	0.0322
	5	1.4363	0.2171	7.9132	0.1611	2.1974	0.0598	12.1165	0.0332	2.1751	0.0658	12.4705	0.0289
	6	1.9404	0.081	13.084	0.0417	2.0783	0.062	14.0283	0.0293	1.7727	0.1169	12.5569	0.0506
	7	1.9326	0.0719	15.518	0.0299	1.3993	0.2139	11.2496	0.1281	1.4355	0.2054	12.2326	0.0932
	8	0.9631	0.4693	9.0277	0.34	1.1916	0.312	11.1861	0.1914	0.9089	0.5147	9.1443	0.3303
	9	1.2592	0.2692	13.5752	0.1383	1.141	0.3422	12.3224	0.1957	0.9741	0.4699	11.4113	0.2486
	10	1.2641	0.2622	15.4956	0.115	1.199	0.3021	14.7273	0.1423	0.7314	0.692	9.8745	0.4516
T2MWET	1	1.9529	0.1648	2.0018	0.1571	12.3812	**0.0006**	12.6933	**0.0004**	11.607	**0.001**	12.0073	**0.0005**
	2	1.0393	0.3569	2.1675	0.3383	3.3771	0.0375	7.0454	0.0295	1.3458	0.2659	2.8518	0.2403
	3	1.9345	0.128	6.1597	0.1041	2.8458	0.0408	9.0662	0.0284	5.5091	0.0017	17.9555	0.0004
	4	2.0043	0.0988	8.6672	0.07	1.685	0.1586	7.2915	0.1213	2.7823	0.0323	12.4131	0.0145
	5	1.8392	0.1112	10.1328	0.0716	1.9057	0.0993	10.5082	0.0621	2.9543	0.0173	16.9382	0.0046
	6	2.3743	0.0343	16.0097	0.0137	1.8296	0.1004	12.3497	0.0546	3.3624	0.0056	23.8169	0.0006
	7	2.4425	**0.0235**	19.6118	**0.0065**	1.1336	0.3482	9.1139	0.2446	2.1655	0.048	18.4541	0.0101
	8	1.2257	0.2921	11.4891	0.1755	1.288	0.2586	12.0912	0.1472	0.7529	0.6449	7.5741	0.4761
	9	1.6754	0.1054	18.0632	0.0344	1.1899	0.3105	12.8512	0.1695	0.8528	0.5713	9.9894	0.3513
	10	1.5624	0.1303	19.1515	0.0384	1.3082	0.2379	16.0686	0.0977	0.6411	0.7728	8.6545	0.5652
TS	1	1.4707	0.2276	1.5075	0.2195	1.5238	0.2195	1.5623	0.2113	0.0007	0.9795	0.0007	0.9791
	2	1.0431	0.3556	2.1754	0.337	1.3522	0.2627	2.8209	0.244	0.8303	0.4395	1.7595	0.4149
	3	1.5904	0.1956	5.0642	0.1672	0.9384	0.4247	2.9896	0.3932	3.7509	**0.0141**	12.2253	**0.0067**
	4	1.2398	0.2983	5.3611	0.2522	1.1013	0.3597	4.7658	0.3122	3.2266	0.0167	14.3955	0.0061
	5	1.5533	0.1795	8.5576	0.1281	0.8244	0.535	4.5457	0.4738	2.392	0.0455	13.7141	0.0175
	6	2.2943	0.0403	15.4702	0.0169	0.6714	0.673	4.5317	0.6051	2.3456	0.0399	16.6143	0.0108
	7	2.602	**0.0164**	20.8926	**0.0039**	0.9914	0.4419	7.9706	0.3352	2.4228	0.028	20.6467	0.0043
	8	1.1825	0.3173	11.0842	0.197	2.19	**0.0345**	20.5591	**0.0084**	1.289	0.2646	12.968	0.113
	9	1.5948	0.1276	17.1942	0.0458	1.9168	0.0586	20.7013	0.014	1.0791	0.3906	12.6409	0.1795
	10	1.3086	0.2375	16.0403	0.0985	1.6422	0.107	20.1709	0.0277	0.8741	0.5618	11.7999	0.2987

Dew point temperature (T2MDEW) and wet-bulb temperature (T2MWET) showed significant results across several lags, with the most consistent significance observed during the second and third pandemic waves at lag 1 (with p-values <0.006). Surface temperature (TS) demonstrated significant results at lag 7 in the first wave, lag 8 in the second wave, and lag 3 in the third wave.

These findings highlight the significant association of air temperature, wet-bulb temperature, dew point temperature, and surface temperature with the spread of COVID-19 across the pandemic waves.

### 4.4 Comparison of proposed model with other modeling

The proposed model facilitates the utilization of available ECDs to better understand their complex associations with the occurrence patterns of infectious diseases. Table 7 shows a comparison of the proposed model with the other correlation analysis modeling.

**Table 7:** Comparison of existing correlation analysis with the proposed approach for COVID-19 pandemic.

S.No.	Author, Reference No.	Study Period-Area	No. of ECDs used for Analysis	Method Used	Associated Parameters Identified
1	Xie and Zhu (2020), [22]	Jan 23, 2020 to Feb 29, 2020 - The majority of the Chinese mainland	3	Spearman Correlation, Generalized additive model	Nonlinear relationship between ambient temperature, positive linear relationship with mean temperature.
2	Kumar et al. (2020), [25]	April 27 to July 25, 2020 - India	4	Spearman rank correlation, Artificial Neural Network	Temperature, Wind Speed, Relative humidity and Pressure
3	Melo et al. (2020), [23]	Dec 2019 to April 6, 2020 - USA	5	Vector Autoregressive (VAR) model, Granger causality test	Temperature with the lag of seven days
4	Muktavat et al. (2021), [30]	Jan 3, 2021 -May 15, 2021 - India	4	Kendall rank correlation and Spearman rank correlation tests at 95 % significance	Positive correlation with temperature; Negative correlation with humidity and AQI
5	Jana et al., (2023), [31]	April 26, 2020 to July 10, 2021 - India	3	Bivariate spatial association	Aerosol Optical Depth (AOD), O_3_, and NO_2_
6	Tyagi et al., (2024), [32]	Apr 1, 2020 to November 12,2020 - India	5	Autoregressive distributed lag models	PM2.5 and PM10 levels, temperature, humidity and wind speed
7	Balasubramani et al. (2024), [29]	Wave I: April 2020 to Jan 2021 Wave II: Feb 2021 to August 2021 - India	4	Negative Binomial Regression Model	Wind speed in wave II, PM_2.5_ in wave I and wave II (for incidence and mortality), Rainfall (for mortality) Key indicators – wealth index (based on occurrence and death rate in wave I and wave II) and Literacy Rates in Wave II (for incidence and mortality) both wave I and II (for mortality), house hold density and overcrowded house hold (for incidence and mortality)
8	**Proposed**	**Jan 3, 2020 to Oct 31, 2022 - India**	**31**	**Convergent Search – Add or Eliminate (CS-AE) Algorithm**	**Determinant of Concern:** Shortwave Downward Radiation (SDR) **Determinant of Interest:** EVI, Photosynthetically Active Radiation, Humidity, Soil Moisture, Surface Pressure, Temperatures, Longwave Downward Radiation, UVA radiation, UVB Radiation

When compared with the existing methodology, the proposed CS-AE algorithm analyzed the maximum number of ECDs and facilitated the categorization of ECDs of greater concern. This categorization highlights the importance of analysing ECDs in the occurrence patterns of infectious diseases. Classifying ECDs as DOC and DOI can provide a framework for developing Climate-Resilient Infectious Disease strategies, which serve as the foundational step for SPRS.

### 4.5 Sustainable pandemic response strategy (SPRS)

The development of SPRS is based on the identification of environmental risk factors, prioritizing key risk factors, integrating environmental insights and evaluating the impact. The proposed CS-AE algorithm helps development of standard operational framework for the sustainable pandemic management for the COVID-19 through categorization of the concern ECDs for an infectious disease. For COVID-19 occurrence, CS-AE algorithm gave following insights:

1. Shortwave Downward Radiation is identified as a DOC. Twenty ECDs have been categorized as DOIs.
2. Analysis of the insights from this identification have been made using Radar Chart Visualization, which helped illustration of the complex relationships between each ECD and the occurrence of COVID-19 in India. This also revealed three distinct spikes, corresponding to the three different waves of the pandemic in India
3. All correlation cannot be a causation, with the analysis of the cause and effect study using the Granger Causality Test. It is identified that for SDR, and interconnected components of Earth's climate systems shows a significant *p* value (*p* < 0.003).

#### 4.5.1 Advantage and limitation of CS-AE algorithm

Hence, the CS-AE algorithm can contribute to the development of a SPRS by providing valuable insights into the relationship between COVID-19 occurrence in India and ECDs. This approach can also be applied to the study of other infectious disease occurrence patterns, provided that there are high-quality time series datasets available. The CS-AE algorithm is applicable only if the datasets are of high quality and contain time series data. Otherwise, it may lead to underfitting, overfitting, or erroneous identification.

#### 4.5.2 Infectious disease control policy

Effective control of infectious diseases requires a collaborative approach involving public health, environmental, and weather monitoring departments. Earth Observation data can be utilized to study ECDs that may influence the occurrence patterns of infectious diseases. By integrating historical infectious disease data with real-time environmental monitoring, patterns can be identified to develop predictive models. Advanced approaches like the CS-AE algorithm can categorize ECDs as DOC and DOI, assessing their impact on various infectious diseases, thereby enabling targeted interventions. A centralized dashboard can be implemented to continuously monitor ECDs alongside past disease occurrences, providing an early warning system for potential outbreaks. This real-time surveillance allows authorities to anticipate pandemic waves, allocate resources efficiently, and implement timely precautionary measures. By leveraging environmental monitoring and predictive analytics, governments and public health officials can enhance preparedness, reduce disease transmission risks, and mitigate the impact of future outbreaks.

## 5 Conclusion

The proposed CS-AE algorithm has ability to categorize ECDs as Determinant of Interest (DOIs) and Determinants of Concern (DOCs) playing a crucial role in understanding the spread of infectious diseases such as COVID-19. Analysis of the correlation between these ECDs and COVID-19 occurrences help the CS-AE algorithm not only in highlighting the significant factors but also provides insights into the complex interdependencies within Earth's climate system.

One of the pivotal findings from the CS-AE algorithm with respect to COVID-19 spread is the identification of Shortwave Downward Radiation (SDR) as a DOC. SDR serves as a key determinant with strong influence on other ECDs, such as air temperature, surface temperature, vegetation index, humidity etc., It plays a significant role in shaping atmospheric dynamics, and its interaction with these climate components creates intricate feedback loops. Changes in SDR can trigger ripple effects across these interconnected climate elements, with significant interpret on the spread of COVID-19. This confirms the vital role of temperature dynamics, including air, wet, dew, and surface temperature, in the occurrence patterns of infectious diseases.

Performance of Granger Causality test was done for the identification of the causation behind the correlation. It showed SDR Granger-causes COVID-19 spread at lags of 6 and 7 days, while the later waves showed a shorter lag of 3 days. Hence the proposed CS-AE algorithm proved to be a powerful tool for identification and the analysis of the interplay between climate determinants and infectious disease occurrences. Its application can help provision of crucial insights for the management of current and future pandemics through integration of environmental risk factors into disease control policies. However, it is important to note that, high-quality time series datasets are crucial to ensure effectiveness for this methodology. In the absence of such datasets, issues like underfitting or overfitting could compromise the validity of the results.

In conclusion, the traditional statistical correlation based on multivariate correlation analysis, identified air temperature as an indirect association with the COVID-19 spread. However, the proposed CS-AE statistical-based evolutionary algorithm revealed, SDR as a more significant determinant compared to other determinants. Also, SDR is shown as significant in altering other ECDs. This indicates the proposed approach as, uncovering detailed interactions with the offer of deeper insights into the investigation of ECDs in relation to infectious diseases. It suggests monitoring SDR for disease transmission patterns especially during outbreak/pandemic could indeed help in framing more effective public health policies. More targeted and effective strategies can be developed for mitigation of the infectious diseases, both now and in the future with the use of the CS-AE algorithm.

## CRediT authorship contribution statement

**Jaraline Kirubavathy K:** Writing – review & editing, Writing – original draft, Visualization, Validation, Software, Resources, Project administration, Methodology, Investigation, Formal analysis, Data curation, Conceptualization. **Thulasi Bai V:** Writing – review & editing, Validation, Supervision, Project administration, Conceptualization.

## Declaration of generative AI and AI-assisted technologies in the writing process

During the preparation of this work, the author(s) used ChatGPT to refine language and improve readability. After using this tool, the authors reviewed and edited the content as needed and takes full responsibility for the content of the published article.

## Declaration of competing interest

The authors have no relevant financial or non-financial interests to disclose.

## Data Availability

The data from this study are available from the corresponding author upon reasonable request.
